# Severity of Vessel Color Changes and Macular and Peripheral Whitening in Malarial Retinopathy Are Associated with Higher Total Body and Sequestered Parasite Burdens

**DOI:** 10.3390/tropicalmed9110279

**Published:** 2024-11-16

**Authors:** Chiadika Nwanze, Daniel Muller, Priscilla Suleman, Mrinmayee Takle, John R. Barber, Kyle J. Wilson, Nicholas A. V. Beare, Karl B. Seydel, Douglas G. Postels

**Affiliations:** 1 Department of Pediatrics, Children’s National Hospital, Washington, DC 20010, USA; chiadika.nwanze@childrens.harvard.edu; 2 George Washington School of Medicine and Health Sciences, Washington, DC 20052, USA; dmuller95@gwmail.gwu.edu; 3 Blantyre Malaria Project, Kamuzu University of Health Sciences, Blantyre, Malawi; misssuleman98@gmail.com (P.S.); seydel@msu.edu (K.B.S.); 4 Division of Neurology, Children’s National Hospital, Washington, DC 20010, USA; mtakle@childrensnational.org (M.T.); kyle.wilson@liverpool.ac.uk (K.J.W.); 5 Division of Biostatistics and Study Methodology, Children’s National Hospital, Washington, DC 20010, USA; jrbarber@childrensnational.org; 6 Department of Eye & Vision Science, University of Liverpool, Liverpool L7 8TX, UK; n.beare@liverpool.ac.uk; 7 Department of Osteopathic Medical Specialties, College of Osteopathic Medicine, Michigan State University, East Lansing, MI 48824, USA

**Keywords:** malaria, pediatrics, retinopathy

## Abstract

Two-thirds of children with cerebral malaria (CM) exhibit retinopathy characterized by whitening, vessel color changes, and/or hemorrhages. The pathogenesis of malarial retinopathy is not fully understood. This study aimed to assess the relationship between malarial retinopathy and the severity of its components (macular whitening, retinal hemorrhages, and vessel color changes) with the total, circulating, or sequestered parasite load in children with CM. Total parasite burden was estimated by measuring plasma levels of Plasmodium falciparum histidine-rich protein 2 (PfHRP2), while the sequestered load was calculated as the difference between the total burden and circulating parasitemia. Children with retinopathy-positive CM (n = 172) had higher total and sequestered parasite burdens compared to retinopathy-negative children (n = 42) (both *p* = 0.049). In a subgroup with detailed retinopathy grading (n = 52), more extensive vessel color changes correlated with higher total, sequestered, and circulating parasite loads (*p* = 0.0057, *p* = 0.0068, and *p* = 0.0433, respectively). Peripheral retinal whitening was also associated with increased total and sequestered loads (*p* = 0.0017 and *p* = 0.0012). No association was found between retinal hemorrhages and parasite burden, indicating that other factors may influence their pathogenesis.

## 1. Introduction

Malaria remains a significant contributor to death and disability in sub-Saharan Africa [1,2,3]. In 2022, there were an estimated 249 million malaria cases in 85 endemic countries, with 94% of them in sub-Saharan Africa [1]. Infection varies from asymptomatic parasitemia to severe disease with organ dysfunction. Of the severe malaria syndromes, cerebral malaria (CM) carries the highest mortality risk, and survivors are at risk of neurological, cognitive, and behavioral sequelae [3,4]. The burden of CM in Africa is greatest in children less than 5 years old.

Efforts to decrease mortality and morbidity from pediatric CM have been hampered by an incomplete understanding of the pathophysiologic sequence between malaria infection and death or disability. Circulating parasitemia is commonly used to assess disease severity in malaria but is a weak predictor of mortality, as the less pathogenic circulating stages are counted on microscopy [5]. Erythrocyte sequestration, the permanent removal of parasitized erythrocytes from the circulation, is likely important in disease pathophysiology. Children dying of CM have intense sequestration of parasitized erythrocytes in cerebral postcapillary venules [6,7].

Upon schizont rupture, sequestered erythrocytes release histidine-rich protein 2 (HRP2) and other parasite-encoded proteins into plasma, where they are available for quantification. Since there are no validated methods of directly measuring sequestration in living children, indirect methods have been developed to estimate its intensity [8]. The most commonly used method calculates the sequestered parasite load as the difference between the total parasite burden (estimated using plasma levels of biomarkers released at red cell schizogony) and circulating parasitemia.

Malarial retinopathy is defined as the presence of macular or peripheral retinal whitening, changes in vessel color, and/or retinal hemorrhages on direct or indirect ophthalmoscopy [9]. Two-thirds of children with CM are retinopathy-positive [10]. On ophthalmoscopy of children with CM, vessel color changes are a visualization of sequestered erythrocytes. Autopsy studies of children dying of retinopathy-positive CM confirm sequestered parasitized erythrocytes within the retinal vessels, supporting the hypothesis that vessel color changes are due to sequestration [11,12]. Retinal whitening is likely an ischemic change with reduced perfusion causing oncotic changes to the cells of the inner retina. This hypothesis is supported by fluorescein angiogram studies showing a correspondence between areas of capillary non-perfusion and areas of pallor on retinal exam [11,13,14]. The pathophysiology of retinal hemorrhages is less clear, although they may indicate a temporary rupture of the blood–retina barrier; their severity is associated with both fatal brain swelling and the severity of brain microhemorrhages in children dying of CM [15].

This study aimed to evaluate whether the three components of malarial retinopathy, retinal whitening, retinal hemorrhages, or vessel color changes, were associated with sequestration intensity in pediatric CM. To evaluate this possibility, the association between malarial retinopathy and between the constituent components of retinopathy, with total body circulating or sequestered parasite loads, was assessed in children with CM.

## 2. Materials and Methods

### 2.1. Clinical Care and Specimen Collection

Children ages 6 months to 13 years old with a clinical diagnosis of CM, defined as a Blantyre coma scale (BCS) ≤ 2, *Plasmodium falciparum* parasitemia, and no alternative explanation for illness, and admitted to Queen Elizabeth Central Hospital in Blantyre, Malawi between January 2010 and March 2023, were included. All children were hospitalized in a unit specialized in the care of children with CM. Clinical care included intravenous antimalarials, antiseizure medications for both clinical and non-convulsive seizures, and close monitoring of blood glucose.

To determine malarial retinopathy status, children underwent dilated direct and indirect ophthalmoscopy by clinician experts in the assessment of retinal changes. In a subset of included children, the individual components of malarial retinopathy were assessed by ophthalmologists using a standard grading system [16]. In each eye, ophthalmologists assessed the number of hemorrhages, estimated the area of whitening in the macula compared to the size of the optic disc, and described the number of quadrants with peripheral whitening and vessel changes [16]. The severity of peripheral whitening was also graded.

Blood was drawn at admission and centrifuged for 10 min at 1500× *g*. The supernatant was pipetted into a cryovial and archived at −80 °C. Concurrently, thick and thin blood smears were prepared for quantitation of circulating parasitemia [9]. A full blood count was collected and analyzed in the hospital laboratory.

Ethical approval for the prospective parent study was provided by the University of Malawi College of Medicine (Blantyre, Malawi) Research Ethics Committee and Michigan State University (East Lansing, MI, USA). At the time of enrollment into the parent observational study, families consented to the use of de-identified biological samples and clinical data, as were used in this analysis. This secondary analysis received ethical approval from the Children’s National Hospital (Washington, DC, USA).

### 2.2. Laboratory Analyses

The circulating parasite density was determined by counting the number of parasites against 200 white blood cells (WBCs) with two tally counters on the thick smear under the light microscope with a 100× magnification lens. Technicians counted parasites on a thin film if 100 parasites or more were observed in every field. On the thin film, infected red blood cells (RBCs) were counted against up to 500 total RBCs. We calculated the circulating parasitemia using either RBC or WBC counts, as determined by a full blood count drawn on admission and analyzed on a Coulter AC.T5diff AL (Beckman Coulter Life Sciences, Indianapolis, IN, USA). The total circulating peripheral load was calculated using the parasitemia calculated from a peripheral blood smear at the time of admission and a calculation of the total body blood volume based on 80 mL of blood/kg body weight [17].

To estimate the total body parasite burden, plasma levels of *Plasmodium falciparum* histidine-rich protein 2 (PfHRP2) were determined. PfHRP2 is restricted to *P. falciparum* and is abundant in the blood stage of infection with persistence beyond parasite clearance. To analyze the plasma concentrations of PfHRP2 levels, commercially available ELISA kits were used (Cellabs, Brookvale, Australia). Manufacturer protocols were followed except that incubations were carried out at 37 °C. Patient plasma samples were diluted at a range of 1:2–1:1000 in 0.1% bovine serum albumin/phosphate-buffered saline (PBS) to produce OD levels in a linear range.

### 2.3. Statistical Analyses

The sequestered parasite load was estimated by determining the total body parasite burden (as established by the results of the PfHRP2 enzyme-linked immunosorbent assay, detailed above) and subtracting the circulating parasite load [8].

Demographic and clinical characteristics between children who were retinopathy-positive and retinopathy-negative were compared using t-tests for continuous data that were normally distributed, Wilcoxon rank sum tests for continuous data that were not normally distributed, or the chi-square/Fisher’s exact tests for categorical data. Circulating, total, and sequestered parasite burdens in those who were retinopathy-negative to children who were retinopathy-positive and to each of the individual components of malarial retinopathy were compared using the same tests.

To assess the association between the severity of the individual components of malarial retinopathy with the total, circulating, and sequestered parasite loads, the raw retinopathy data were transformed into an average severity score. The severity of vessel changes or peripheral whitening was calculated in both the right and left eye by taking the number of retinal quadrants in which vascular changes or whitening were seen and dividing by the number of quadrants visualized. Further, peripheral retinal whitening was graded from 1 to 3, with a grade of 1 representing scattered whitening, a grade of 3 with confluent whitening, and a grade of 2 as intermediate. We took an average across both eyes. The severity of macular whitening was graded as the number of areas of the optic disc of whitening seen, averaged across both eyes. The severity of hemorrhages was assessed by determining the mean number of hemorrhages across both eyes. In all cases, they were divided by the number of retinal quadrants visualized. After the severity score for the individual retinopathy component was obtained, simple linear regression was used to assess the association between the severity of each retinopathy component and the intensity of the total, circulating, and sequestered parasite burdens.

In all analyses, tests were 2-sided and a *p*-value less than 0.05 was considered a statistically significant difference between groups.

## 3. Results

During the 13-year enrollment period, 262 children with CM were admitted to the hospital and had biological samples available. Of these, 157 had received two or more doses of intravenous antimalarials before hospital admission. Data from these children were excluded, as it was assumed that parasite clearance had begun, making their circulating parasite loads unreliable. Children whose data were excluded were more likely to survive with neurologic sequelae, but there were no other significant differences between those whose data were or were not included (Table 1).

Of the 105 children included in all analyses, there were no statistically significant differences in the Blantyre coma scale, circulating parasitemia, or outcomes when comparing children with and without malarial retinopathy (Table 2). Of the 91 children with cerebral malaria who were retinopathy-positive, 70 (76.9%) had retinal whitening, 63 (69.2%) had retinal hemorrhages, and 55 (60.4%) had vessel color changes.

When total body parasite burden was estimated using PfHRP-2, children with retinopathy-positive CM had higher total body and sequestered parasite burdens (both *p* = 0.049) compared to those who were retinopathy-negative (Table 3). There was no association between the circulating parasite load and the presence of malarial retinopathy.

**Figure 1:**
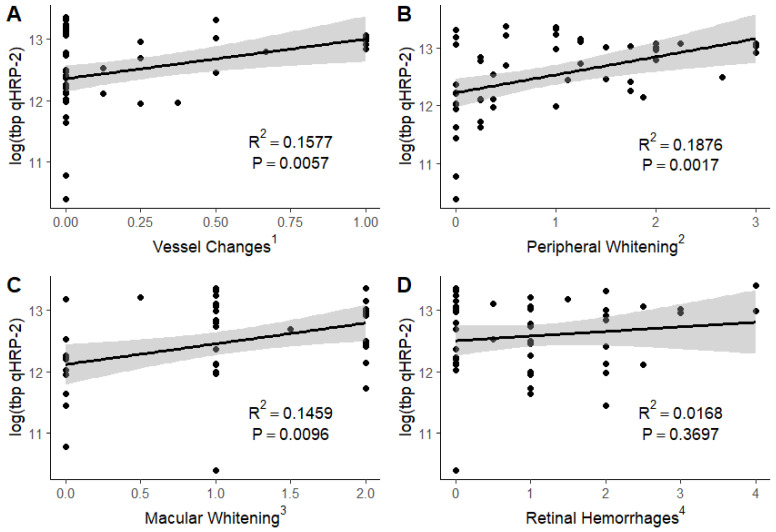
Association of total body parasite burden with severity of vessel color changes (**A**), peripheral whitening (**B**), macular whitening (**C**), and retinal hemorrhages (**D**). log (tbp qHRP-2): logarithm of total body parasite burden as determined by analysis of blood quantitative histidine-rich protein 2. ^1^ Vessel changes are graded as no (0) or yes (1) in each quadrant of both eyes, divided by the number of quadrants visualized, with a mean taken between the eyes. ^2^ Peripheral whitening is graded as absent (0), mild (1), moderate (2), or severe (3). Grade 3 peripheral whitening must be a widespread mosaic or have patches of confluence. ^3^ Macular whitening is graded as absent (0), less than one-third disc (optic nerve) area in diameter (1), one-third to one disc area in diameter (2), greater than one disc area in diameter (3), with a mean taken between the two eyes. ^4^ Retinal hemorrhages are graded as absent (0), 1–5 hemorrhages (1), 6–20 hemorrhages (2), 21–50 hemorrhages (3), or 51 or more hemorrhages (4).

Linear regression analyses of the severity of the individual components of retinopathy with total, circulating, and sequestered parasite burdens were limited to the 48 children who had detailed standardized grading of the severity of vessel color changes, hemorrhages, and macular and peripheral whitening by ophthalmologists. When the total body parasite burden was estimated using PfHRP2, we found significant positive associations between vessel changes (R^2^ = 0.1577; *p* = 0.0057), peripheral whitening (R^2^ = 0.1876; *p* = 0.0017), and macular whitening (R^2^ = 0.1459; *p* = 0.0096) but not hemorrhages (R^2^ = 0.0168; *p* = 0.3697) with the total body parasite burden (Figure 1).

**Figure 2:**
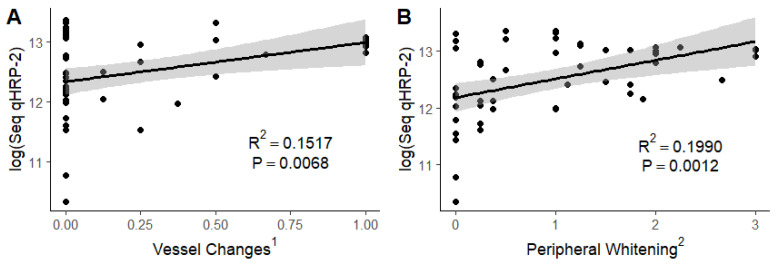

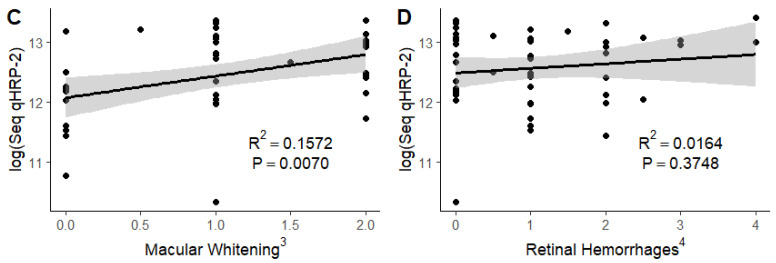
Association of sequestered parasite burden with severity of vessel color changes (**A**), peripheral whitening (**B**), macular whitening (**C**), and retinal hemorrhages (**D**). Log (seq qHRP-2): logarithm of sequestered parasite load as determined by estimating total body parasite burden using quantitative histidine-rich protein 2 and subtracting circulating parasite burden. ^1^ Vessel changes are graded as no (0) or yes (1) in each quadrant of both eyes, divided by the number of quadrants visualized, with a mean taken between the eyes. ^2^ Peripheral whitening is graded as absent (0), mild (1), moderate (2), or severe (3). Grade 3 peripheral whitening must be a widespread mosaic or have patches of confluence. ^3^ Macular whitening is graded as absent (0), less than one-third disc (optic nerve) area in diameter (1), one-third to one disc area in diameter (2), greater than one disc area in diameter (3), with a mean taken between the two eyes. ^4^ Retinal hemorrhages are graded as absent (0), 1–5 hemorrhages (1), 6–20 hemorrhages (2), 21–50 hemorrhages (3), or 51 or more hemorrhages (4).

We found significant positive associations between vessel changes (R^2^ = 0.1577; *p* = 0.0068), peripheral whitening (R^2^ = 0.1990; *p* = 0.0012), and macular whitening (R^2^ = 0.1572; *p* = 0.0070) but not with retinal hemorrhages (R^2^ = 0.0164; *p* = 0.3748) when the severity of the constituent component was regressed on the sequestered parasite burden (Figure 2).

**Figure 3:**
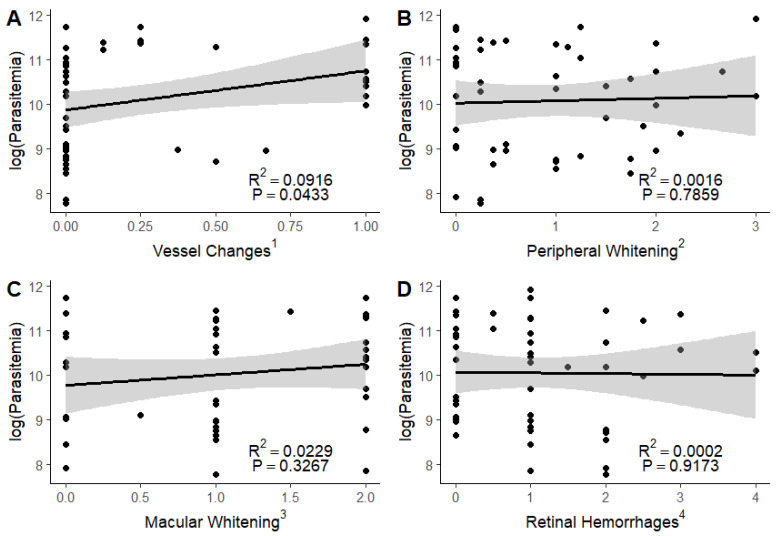
Association of circulating parasite burden with severity of vessel color changes (**A**), peripheral whitening (**B**), macular whitening (**C**), and retinal hemorrhages (**D**). Log (parasitemia): logarithm of circulating parasite burden. ^1^ Vessel changes are graded as no (0) or yes (1) in each quadrant of both eyes, divided by the number of quadrants visualized, with a mean taken between the eyes. ^2^ Peripheral whitening is graded as absent (0), mild (1), moderate (2), or severe (3). Grade 3 peripheral whitening must be a widespread mosaic or have patches of confluence. ^3^ Macular whitening is graded as absent (0), less than one-third disc (optic nerve) area in diameter (1), one-third to one disc area in diameter (2), greater than one disc area in diameter (3), with a mean taken between the two eyes. ^4^ Retinal hemorrhages are graded as absent (0), 1–5 hemorrhages (1), 6–20 hemorrhages (2), 21–50 hemorrhages (3), or 51 or more hemorrhages (4).

We found a significant association between circulating parasitemia and vessel changes (R^2^= 0.0916, *p* = 0.0433) but none of the other features of malarial retinopathy (Figure 3).

## 4. Discussion

In children with malarial retinopathy, the severity of macular and peripheral whitening and vessel color changes is associated with increasing total body and sequestered parasite burdens, estimated using quantitative PfHRP2. There was no association found between the severity of retinal hemorrhages and parasite burden.

The hallmark of *Plasmodium falciparum* malaria infection, and the pivotal event in the pathogenesis of severe malaria, is the sequestration of infected red blood cells in postcapillary venules and other small vessels [7]. This process facilitates parasite evasion to avoid filtration of mature parasite stages by the spleen. Severe malaria is characterized by the sequestration of mature stages of asexual *Plasmodium falciparum* in the capillaries and venules of vital organs, making blood parasitemia an inaccurate marker of severe malaria severity.

PfHRP2 is a parasite-encoded protein released at erythrocyte schizogony and can be quantitated to estimate the total body parasite burden [18]. The function of PfHRP2 in the pathogenesis of severe malaria is poorly understood, but it is thought to promote cell adherence to the endothelial surface, affecting blood flow, blood–brain barrier integrity, and avoidance of splenic clearance [19].

Rates of PfHRP2 deletion in malaria parasites in Malawi are currently low [20]. Should rates of these deletions increase in the parasite population, the presence of retinopathy (and its severity) in a febrile comatose child could be used to suggest that malaria is responsible for illness despite a negative PfHRP2 rapid diagnostic test. Although these analyses found an association between both the total body and sequestered parasite burdens and the severity of malarial retinopathy, we do not advocate using malarial retinopathy (a subjective measure) to estimate parasite loads an objective one. In geographic locations with emerging rates of HRP2 deletions, we believe that the development and clinical implementation of alternative, non-HRP2-based malaria rapid diagnostic tests should be prioritized. Since the current methods of determination of both the total body and sequestered parasite burdens are based on quantitative determinations of HRP2, as deletions within the parasite population increase in prevalence, alternative methods for estimation of these burdens will be needed.

### Study Advantages and Limitations

This study has several advantages. Clinicians who collected the biospecimens and provided clinical care varied little during the 13-year data collection period, decreasing the likelihood of variations through time and producing differences in outcomes. Laboratory analyses were standardized and performed in duplicate, decreasing the risk of measurement errors.

These analyses have several limitations. Most importantly, treatment with antimalarial medications prior to hospitalization, which were used to exclude patients whose peripheral parasite densities may have been rapidly decreasing, may not have been completely documented. It is possible that some children were included in these analyses who may have received two or more doses of intravenous antimalarials, making their circulating parasite loads dynamic and changing the study’s results. Conversely, some children may have been eliminated from the analyses who should not have been. It is possible that some children had antimalarial administration documented on their pre-hospital paperwork yet did not receive it. Due to high rates of pre-hospital treatment with broad-spectrum antimalarials, circulating parasitemia in our patients was very low. Therefore, the total body and sequestered parasite loads were little different from one another (Table 3). In Malawi, where public health measures have pushed out malaria rapid diagnostic tests and broad-spectrum antimalarials to peripheral health centers, children almost always arrive at our referral center having received at least one dose of an antimalarial. Future studies may consider restricting enrollment to children who have received no pre-hospital antimalarial treatment. The number of children with detailed retinal exams (and grading of the individual retinopathy components) was few. This may have limited the study’s power to establish a statistically significant difference between groups. Finally, the determination of malarial retinopathy status and its grading is a subjective process, and only one ophthalmologist examined patients. Consensus determinations of malarial retinopathy status and its severity by multiple ophthalmologist examiners may have changed our study’s findings.

## 5. Conclusions

In summary, when the total body parasite burden is estimated using PfHRP2, the presence of malarial retinopathy and the severity of either vessel color changes, macular whitening, or peripheral whitening is associated with increasing intensity of both the total body and sequestered parasite burdens. No association between retinal hemorrhages and parasite burden was found, suggesting other factors are involved with their pathogenesis.

## Figures and Tables

**Table 1:** Characteristics of children with complete retinopathy data who were included in analyses (were not pre-treated, n = 105) to those who were excluded (were pre-treated, n = 157).

		Included in Analyses (n = 105)	Excluded from Analyses Due to Receipt of 2 or More Doses of parenteral Antimalarials Pre-Hospital (n = 157)	*p*-Value for Difference
Age (months): mean, SD		54.7 (30.9)	54.2 (29.7)	0.908
Sex: n (%) male		56 (53%)	91 (58%)	0.495
Blantyre coma score: n (%)				
	0	11 (10%)	14 (9%)	0.948
	1	39 (37%)	59 (37%)	
	2	55 (52%)	84 (53%)	
Malarial retinopathy status: n (%) positive		91 (87%)	102 (65%)	<0.001
Outcomes: n (%)				0.021
	Died	17 (16%)	20 (13%)	
	Survived and normal	85 (81%)	118 (75%)	
	Survived with neurologic sequelae	3 (3%)	19 (12%)	

SD: standard deviation.

**Table 2:** Demographic, clinical, and laboratory characteristics of included participants.

		Retinopathy-Positive (n = 91)	Retinopathy-Negative (n = 14)	*p*-Value for Difference
Age (months): mean, SD		52.8 (30.6)	66.7 (31.5)	0.118
Sex: n (%) male		49 (54%)	7 (50%)	0.788
Weight (kg): mean, SD		14.0 (5.1)	16.9 (8.1)	0.212
Blantyre coma score: n (%)				
	0	9 (9%)	2 (14%)	0.644
	1	33 (36%)	6 (43%)	
	2	49 (54%)	6 (43%)	
Parasitemia [log (parasites/µL)]		4.0 (1.2) n = 89	3.7 (1.3) n = 13	0.464
Hematocrit (%): mean, SD		22.1 (7.6)	24.2 (2.9)	0.078
Glucose (mmol/L): mean, SD		5.9 (2.2) n = 80	5.6 (2.5) n = 13	0.669
Lactate (mmol/L); mean, SD		6.0 (5.3) n = 90	4.7 (4.5) n = 13	0.258
Outcomes: n (%)				0.805
	Died	14 (15%)	3 (21%)	
	Survived and normal	74 (81%)	11 (79%)	
	Survived with neurologic sequelae	3 (3%)	0 (0%)	

SD: standard deviation.

**Table 3:** Comparison of total body and sequestered parasite masses in children of varying malarial retinopathy status, using quantification of PfHRP2 to estimate total body parasite burden.

	Retinopathy-Positive (n = 91)	Retinopathy-Negative (n = 14)	*p*-Value for Difference
Log (Total body parasite burden): mean, SD	12.50 (0.70)	12.00 (0.83)	0.049
Log (circulating parasite load) mean, SD	10.03 (1.17)	9.78 (1.25)	0.564
Log (Sequestered parasite load): mean, SD	12.50 (0.72)	12.00 (0.84)	0.049

SD: standard deviation. PfHRP2: total body parasite burden estimated using measurement of blood quantitative *Plasmodium falciparum* histidine-rich protein 2.

## Data Availability

The datasets generated during the current study are available from the corresponding author upon reasonable request. Clinical and demographic data derived from the parent study are not the property of the authors and cannot be released by them.
